# Identifying factors associated with the discharge of male State patients from Weskoppies Hospital

**DOI:** 10.4102/sajpsychiatry.v23i0.1083

**Published:** 2017-12-04

**Authors:** Riaan G. Prinsloo, Andre Swanepoel, Gian Lippi

**Affiliations:** 1 Department of Psychiatry, School of Medicine, Faculty of Health Sciences, University of Pretoria, South Africa; 2 Department of Statistics, Faculty of Natural and Agricultural Science, University of Pretoria, South Africa

## Abstract

**Background:**

Designated psychiatric facilities are responsible for the care, treatment and reintegration of State patients. The necessary long-term care places a considerable strain on health-care resources. Resource use should be optimised while managing the risks that patients pose to themselves and the community. Identifying unique factors associated with earlier discharge may decrease the length of stay. Factors associated with protracted inpatient care without discharge could identify patients who require early and urgent intervention.

**Aim:**

We identify socio-economic, demographic, psychiatric and charge-related factors associated with the discharge of male State patients.

**Methods:**

We reviewed the files of discharged and admitted forensic State patients at Weskoppies Psychiatric Hospital. Data were captured in an electronic recording sheet. The association between factors and the outcome measure (discharged vs. admitted) was determined using chi-squared tests and Fischer’s exact tests.

**Results:**

Discharged State patients were associated with being a primary caregiver (*p* = 0.031) having good insight into illness (*p* = 0.025) or offence (*p* = 0.005) and having had multiple successful leaves of absences. A lack of substance abuse during admission (*p* = 0.027), an absence of a diagnosis of substance use disorder (*p* = 0.013) and the absence of verbal and physical aggression (*p* = 0.002 and *p* = 0.016) were associated with being discharged. Prolonged total length of stay (9–12 years, *p* = 0.031) and prolonged length of stay in open wards (6–9 years, *p* = 0.000) were associated with being discharged. A history of previous offences (*p* = 0.022), a diagnosis of substance use disorder (*p* = 0.023), recent substance abuse (*p* = 0.018) and a history of physical aggression since admission (*p* = 0.017) were associated with continued admission.

**Conclusion:**

Discharge of State patients is associated with an absence of substance abuse, lack of aggression, multiple successful leave of absences and length of stay in hospital.

## Introduction

State patients (SPs) are individuals who have been charged with a serious offence (e.g. murder, rape or assault) and are diagnosed with a mental disorder or intellectual disability after a period of psychiatric observation and assessment in terms of Sections 77, 78 and 79 of the *Criminal Procedure Act* (CPA) No. 51 of 1977, where they were found to be unable to follow court proceedings and/or contribute meaningfully to their defence and/or unable to appreciate and/or act in accordance with the wrongfulness of their offence.^1^ These patients are detained in designated psychiatric facilities for further care, treatment and rehabilitation under Section 42 of the *Mental Health Care Act* (MHCA) No. 17 of 2002.^2^

Internationally, the admission of SPs or forensic mental health patients has increased. In the United Kingdom (UK), forensic mental health admissions have increased from the late 1990s to almost 4000 in 2007.^3^ Similar trends have been observed in South Africa. In the Western Cape, SP admissions have increased by nearly 400%, from 205 in 1997 to 800 in 2013. This constituted an annual admission rate of 50 patients with a discharge rate of only 5 per year.^4^ Most South African psychiatric facilities designated for SPs are exceeding their maximum capacity.^5^ Prolonged inpatient admission comes at a significant financial and human resources cost. Alleviating this burden requires limiting the duration of inpatient care without compromising patient or community safety.

Limiting inpatient care requires identifying factors that predict earlier successful reintegration and eventual discharge. Gran et al. developed the Structured Outcome Assessment and Community Risk Monitoring (SORM) to monitor these factors for patients who were conditionally discharged.^6^ Patients are assessed every 30 days during the 2 years after discharge. Although intensive monitoring promotes patient-centred care, SORM may not be effective in low-resource settings. Irrespective of setting, SPs are more likely to be readmitted if they are violent towards others or self, prone to substance abuse and have no social support. These factors were identified in the Midlands (UK) from a limited retrospective case note analysis.^7^ In the Free State, South Africa, a profile of SPs admitted between 2004 and 2008 was constructed from demographic, psychiatric and criminal information.^8^ Major mental illness, substance abuse, severity of the crime, compliance to medication, psychiatric history and support from family and friends were deemed important factors with potential to influence rehabilitation and discharge.^8^ This profiling study did not compare discharged patients with currently admitted patients. In Gauteng, South Africa, Marais and Subramaney conducted a 3-year follow-up study of 114 SPs admitted in 2004 and 2005, of which one-third remained as inpatients and 69% were in the community.^9^ Continued inpatient care was because of risk of re-offense, current mental state and poor family contact.^9^ This study was limited to patients who were admitted as SPs at the time, even though two-thirds were being treated on an outpatient basis.

Our research aims to identify factors that may be associated with the discharge of male SPs from Weskoppies Psychiatric Hospital. We compare currently admitted SPs with discharged SPs and identify factors unique to each group.

## Method

### Study design

We conducted a quantitative, comparative cross-sectional review of patient files. All data were treated anonymously.

### Study population

**FIGURE 1:**
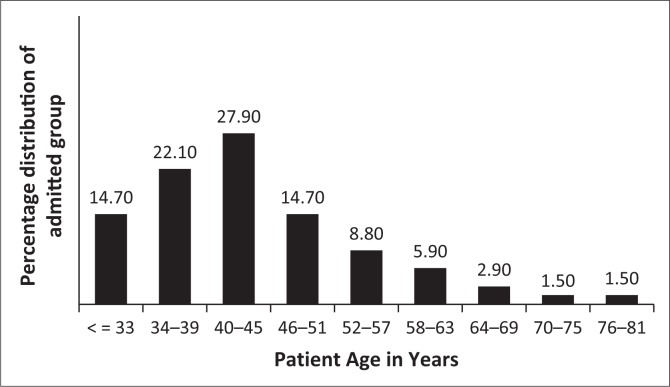
Age distribution for admitted patients.

**FIGURE 2:**
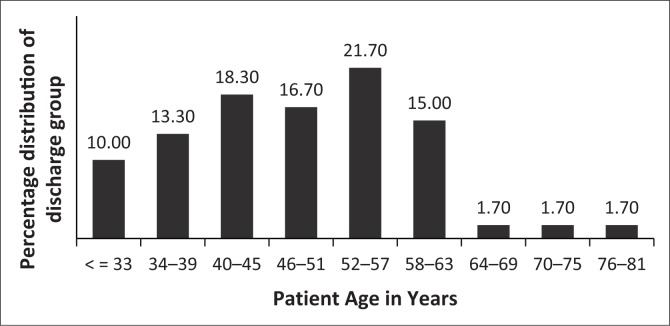
Age distribution for discharged patients.

SPs were divided into two groups, namely those discharged (DG) between 2009 and 2013 and those who were still admitted (AG) in 2015. Initially, 72 discharged male SPs were identified; however, only 68 patients’ files could be traced of which only 60 contained enough information. Of the 212 male SPs still admitted, 68 were randomly selected using age stratification to better compare with the DG. A uniform sample of male SPs was included in the study. Female SPs were not included because of their different patient profiles and small numbers at Weskoppies Hospital. After age stratification, the distribution of age categories did not differ significantly between DG and AG (Fisher’s exact test, *p* = 0.229) (Figures 1 and 2).

### Data collection

Patient files were reviewed and data were captured in a specifically designed data questionnaire. We recorded demographic, socio-economic, psychiatric and offence-related data from each patient’s file. In total, 128 files were reviewed.

### Data analysis

Statistics were performed using SAS software. Data were cross-tabulated using the FREQ procedure. Associations between category (DG, AG) and factors were tested using chi-squared tests (cell frequencies > 5) or Fischer’s exact tests (cell frequencies ≤ 5). Results were statistically significant if *p* < 0.05. The *Z*-test for comparison of proportions determined the statistical significance between the proportions of the two patient groups within a certain category of a factor.

## Results

### Socio-demographic data

Most of the SPs were black people (91.2% AG, 85.0% DG) and single (76.5% AG, 71.7% DG). Having children was not significantly associated with category; however, being the primary care giver was significantly associated with discharge (*p* = 0.035; 0% AG, 6.7.0% DG). Only four SPs were primary caregivers and all four were discharged. Level of education, occupational status, level of premorbid social support and area of origin were not associated with either category. Most of the patients had an incomplete secondary education (AG = 32.4%, DG = 26.7%) followed by an incomplete primary education (AG = 26.5%, DG = 21.7%). Most patients (AG+DG) were unemployed (47.7%) prior to admission.

### Psychiatric history

There was no association between category and previous psychiatric admissions, receiving treatment prior to admission, adherence to treatment prior to the offence or premorbid functioning. Slightly more admitted patients had a history of previous admissions and treatment compared to discharged SPs. Admitted SPs had poorer premorbid functioning. There was no association between category and diagnosis, psychotic disorders, mood disorders, cognitive disorders or mental retardation.

Schizophrenia was the most prevalent diagnosis in both groups (75.0% AG, 60.0% DG), with psychotic disorder not otherwise specified being second most prevalent (8.8% AG, 11.7%DG) (Table 2). Mood disorders and cognitive disorders were not common in the study population. Borderline intellectual functioning was more common amongst the AG (13.2% AG, 3.3% DG), whereas mental retardation, severity unspecified was more prevalent amongst the DG (4.4% AG, 13.3% DG). A diagnosis of antisocial personality disorder (ASPD) was more common amongst admitted SPs (14.7% AG, 5.0% DG). A history of substance use was significantly associated with both AG and DG (*p* = 0.018), yet patients with a history of substance abuse were more common in the AG (77.9% AG, 58.3% DG) and absence of substance use was more common in the DG (19.1% AG, 40.0% DG). Insight into illness was significantly associated with both patient groups (*p* = 0.032). DG patients had higher rates of good insight into their illness (10.3% AG, 26.7% DG). More AG SPs had poor insight into their illness (61.8% AG, 41.7% DG) (Table 2).

**TABLE 1:** Demographic information of male State patients admitted to Weskoppies Hospital (2009 and 2013).

Variables	Admitted *n* (%)	Discharged *n* (%)	*p*
**Race**			**0.302**
Black	62 (91.2)	51 (85.0)	
Caucasian	6 (8.8)	7 (11.7)	
Mixed race	0 (0.0)	2 (3.3)	
**Relationship status**			**0.065**
Single	52 (76.5)	43 (71.7)	
In a relationship	5 (7.4)	3 (5.0)	
Married	2 (2.9)	4 (6.7)	
Separated	2 (2.9)	1 (1.7)	
Divorced	1 (1.4)	8 (13.3)	
Widowed	2 (2.9)	0 (0.0)	
Unknown	4 (5.8)	1 (1.7)	
**Primary caregiver**			**0.035**
Yes	0 (0.0)	4 (6.7)	**0.031**
No	51 (75.0)	36 (60.0)	
**Education level**			**0.108**
No formal education	8 (11.8)	7 (11.7)	
Primary education	18 (26.5)	13 (21.7)	
Completed primary education	10 (14.7)	6 (10.0)	
Secondary education	22 (32.4)	16 (26.7)	
Completed secondary education	6 (8.8)	5 (8.3)	
Tertiary education	1 (1.5)	0 (0.0)	
Completed tertiary education	1 (1.5)	1 (1.7)	
Unknown	2 (2.9)	12 (20.0)	
**Occupation prior to admission**			**0.200**
Employed	6 (8.8)	7 (11.7)	
Informal employment	18 (26.5)	6 (10.0)	
Unemployed	27 (39.7)	34 (56.7)	
Pensioner	3 (4.4)	2 (3.3)	
Disability	8 (11.8)	7 (11.7)	
Unknown	6 (8.8)	4 (6.7)	

Note: Bold values indicate statistically significant results (*p* < 0.05).

**TABLE 2:** Psychiatric history, diagnosis and insight into illness of male State patients.

Variables	Admitted *n* (%)	Discharged *n* (%)	*p*
**Previous psychiatric admissions**			**0.596**
Previously admitted for treatment	29 (42.6)	22 (36.7)	
No previous admissions	23 (33.8)	19 (31.7)	
Unknown	16 (23.5)	19 (31.7)	
**Psychotic disorders**			**0.298**
No psychotic disorder	5 (7.4)	11 (18.3)	
Schizophrenia	51 (75.0)	36 (60.0)	
Schizoaffective disorder, bipolar type	2 (2.9)	3 (5.0)	
Substance induced psychotic disorder	1 (1.5)	2 (3.3)	
Psychotic disorder not otherwise specified	6 (8.8)	7 (11.7)	
**Mood disorders**			**0.669**
Bipolar 1 disorder	1 (1.5)	1 (1.7)	
Personality disorders			**0.113**
Antisocial personality disorder	10 (14.7)	3 (5.0)	
**Substance use disorders**			**0.018**
Substance abuse	53 (77.9)	35 (58.3)	**0.023**
Substance dependence	2 (2.9)	1 (1.7)	
No substance abuse	13 (19.1)	23 (40.0)	**0.013**
**Mental retardation**			**0.054**
Borderline intellectual functioning	9 (13.2)	2 (3.3)	
Mild mental retardation	5 (7.4)	1 (1.7)	
Moderate mental retardation	2 (2.9)	2 (3.3)	
Mental retardation, severity unspecified	3 (4.4)	8 (13.3)	
**Insight into illness**			**0.032**
Good	7 (10.3)	16 (26.7)	**0.025**
Partial	18 (26.5)	16 (26.7)	
Poor	42 (61.8)	25 (41.7)	**0.029**
Unknown	1 (1.5)	3 (5.0)	

Note: Bold values indicate statistically significant results (*p* < 0.05).

### Offence information

**FIGURE 3:**
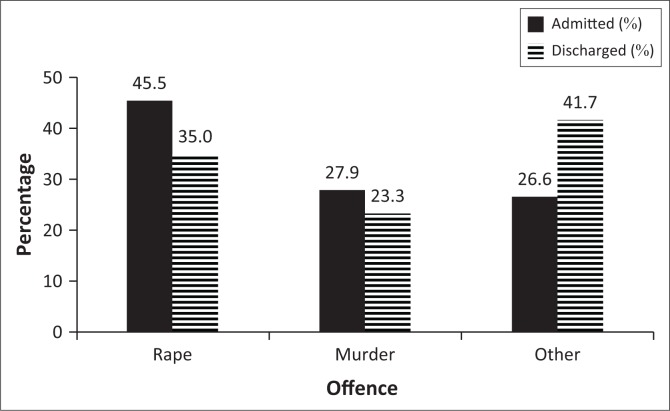
Type of offence.

**FIGURE 4:**
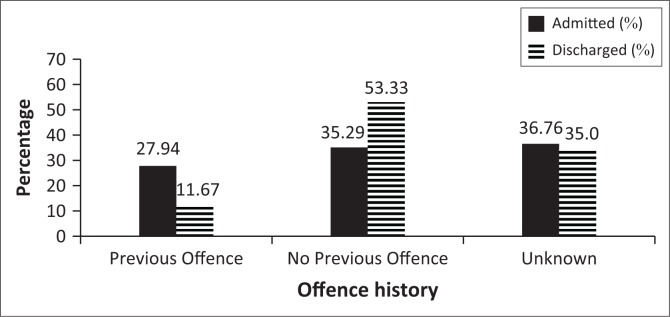
History of previous offences.

**FIGURE 5:**
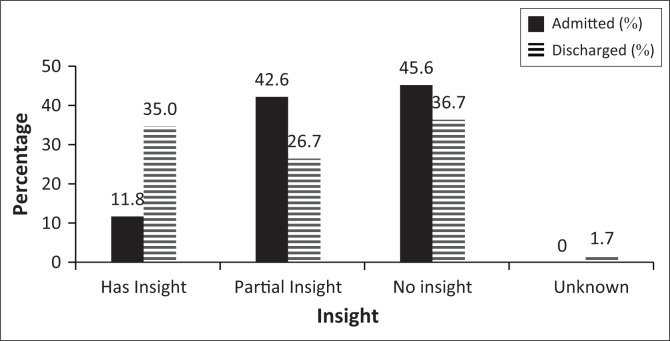
Insight into offence.

Rape and murder were the most prevalent offences, with higher incidences in the AG (rape: 45.6% and murder: 27.9%) compared to the DG (rape: 35.0% and murder: 23.3%) (Figure 3). There was no association between the type of offence committed and the admission status. History of previous criminal offences was statistically significant (*p* = 0.037). A history of a previous offence was associated with the AG (*p* = 0.022; 27.9% AG, 11.7% DG), and there were more DG SPs with no previous offence (*p* = 0.04; 35.3% AG, 53.3% DG) (Figure 4). Insight into offence was associated with category (*p* = 0.005). Twice as many DG SPs showed insight into their offence compared to AG SPs (11.8% AG, 35.0% DG) (Figure 5).

### Duration of admission

Length of stay was measured in 36-month increments. We recorded total length of stay and stay in closed, semi-closed and open wards. The maximum length of stay was 372 months (31 years). Total length of stay (*p* = 0.006) of admitted patients significantly outnumbered those discharged within the first 36 months of admission (*n* = 23, 33.8% AG; *n* = 4, 6.7% DG). The total length of stay of most DG SPs ranged from 109 to 144 months (9–12 years). The DG SPs outnumbered the SPs still admitted for a similar period (11.8% AG, 26.7% DG; *p* = 0.031).

A larger proportion of DG SPs did not spend time in semi-closed wards (19.1% AG, 50.7% DG; *p* = 0.004). Significantly more AG SPs spent less than 36 months in open wards compared to AG SPs (25.0% AG, 18.6% DG; *p* = 0.000). For AG SPs, time spent in open wards declined as length of stay increased. DG numbers in open wards gradually increased and peaked between 73 and 108 months stay (4.4% AG, 27.1% DG; *p* = 0.000) after which they declined but remained higher than the AG. Compared to the DG, a large number of AG SPs have never stayed in an open ward (52.9% AG, 6.8% DG; *p* = 0.000). Successful leaves of absence (LOAs) were categorised in increments of 20 with the highest number of LOAs being 80. More AG than DG SPs have never had successful LOAs (41.18% AG, 13.33% DG; *p* = 0.000).

### Behavioural history

AG SPs were associated with a history of verbal aggression (*p* = 0.012), physical aggression (*p* = 0.038) and substance abuse (0.028) prior to admission (Table 3). DG SPs were associated with the absence of substance abuse (*p* = 0.027), verbal (*p* = 0.002) and physical aggression (*p* = 0.028) during admission (Table 3). AG SPs were associated with physical aggression during admission (*p* = 0.017) (Table 3). Number of attempts at absconding was equally distributed between both groups. There was no association between adherence to treatment and group, although the DG SPs with good adherence outnumbered those in the AG and the AG SPs with intermittent and poor adherence to treatment outnumbered the DG SPs. Both groups were treated with depot antipsychotics, which may have had a positive effect on adherence.

**TABLE 3:** Behavioural history of male State patients admitted to Weskoppies Hospital.

Variables	Admitted *n* (%)	Discharged *n* (%)	*p*
**Verbal aggression**			**0.012**
None	5 (7.4)	17 (28.3)	**0.002**
Before admission	19 (27.9)	14 (23.3)	
Since admission	37 (54.4)	27 (45.0)	
Recent	3 (4.4)	0 (0.0)	
Unknown	3 (4.4)	2 (3.3)	
**Physical aggression**			**0.038**
None	7 (10.3)	16 (26.7)	**0.016**
Before admission	23 (33.8)	25 (41.7)	
Since admission	32 (47.1)	16 (26.7)	**0.017**
Recent	1 (1.5)	0 (0.0)	
Unknown	5 (7.4)	3 (5.0)	
**Sexually inappropriate behaviour**			**0.033**
None	28 (41.2)	34 (56.7)	
Before admission	17 (25.0)	18 (30.0)	
Since admission	9 (13.2)	6 (10.0)	
Recent	2 (2.9)	0 (0.0)	
Unknown	12 (17.6)	2 (3.3)	**0.010**
**Substance use**			**0.028**
None	14 (20.6)	23 (38.3)	**0.027**
Before admission	18 (26.5)	13 (21.7)	
Since admission	24 (35.3)	22 (36.7)	
Recent	6 (8.8)	0 (0.0)	**0.018**
Unknown	6 (8.8)	2 (3.3)	
**Adherence to medication**			**0.061**
Good	42 (61.8)	44 (73.3)	
Intermittent	22 (32.4)	12 (20.0)	
Poor	3 (4.4)	0 (0.0)	
Unknown	1 (1.5)	4 (6.7)	

Note: Bold values indicate statistically significant results (*p* < 0.05).

## Ethical consideration

Ethics approval was granted by the University of Pretoria, Faculty of Health Sciences Research Ethics committee. Permission to review files was obtained from the Chief Executive Officer of Weskoppies Hospital.

## Discussion

We identify factors associated with the discharge or continued admission of SPs at Weskoppies Hospital. Releasing forensic SPs may timeously alleviate the burden on state mental health services, but doing so prematurely may increase the risk of drug abuse and subsequent recidivism. Successful rehabilitation of SPs is reliant on adequate social support, continued monitoring and therapy concomitant with drug rehabilitation. Comparing profiles of discharged and admitted forensic SPs may isolate important factors that contribute to successful discharge.

At Weskoppies Hospital, the number of AG SPs is three times higher than DG SPs (212 AG, 72 DG) (this study). Successful discharge thus remains a challenge. Discharge was not associated with race or religion. Because of stratification, age was not associated with DG or AG. The age distribution within groups varied slightly; 64% of AG SPs were younger than 45 years and 57% of DG SPs were between and 40 and 63 years old. Both groups had similar numbers of patients older than 64. Younger SPs may have recently been admitted; older SPs may have been admitted, may have received and responded to treatment, and been discharged. SPs older than 64 years may no longer have social support or may be too impaired to function outside of an institution. The ability to function outside of an institution may be determined by educational level and employment. Our inability to identify associations between these factors and discharge may be because of the high number of patients whose educational level was unknown. The relatively low levels of education may affect employment status. Most SPs were either unemployed or informally employed. In Norway, long-term psychiatric admission was associated with being single, socially isolated and uneducated.^10^

South African SPs are negatively influenced by substance use disorders.^9^ SPs at Sterkfontein Hospital are prone to alcohol and cannabis abuse.^9^ We found that substance abuse was associated with continued admission, while SPs who had not abused substances were associated with being discharged. At Weskoppies Hospital, substance abuse was prevalent in both groups (70%, this study). Patients with substance use disorders are more likely to commit violent acts^11^ and are therefore more likely to be incarcerated.^12^ The most common offence committed by SPs was rape, followed by murder. Discharge was not associated with a particular offence. In contrast, Marais and Subramaney reported that violent non-sexual offenses were more common amongst SPs at Sterkfontein Hospital.^9^ Their study included female SPs, who are known to commit a different range of offences.^13^ At Weskoppies Hospital, a history of previous offense was associated with continued admission, and discharge was associated with not having committed a previous offence. Patients with a history of criminal behaviour are often considered as higher risk patients and tend to remain in hospital for longer. SPs with insight into offense were more common in the DG. Improved insight into the wrongfulness of the offence could indicate improved judgement and reduced risk subsequently leading to earlier discharge.

SPs at Weskoppies Hospital were most often diagnosed with schizophrenia, followed by substance abuse disorder. Discharge was not associated with a particular disorder. A study by Xafenias et al. found that the diagnosis of substance use disorder was not a significant factor in the length of hospital stay.^14^ However, it should be noted that this study was conducted in a non-forensic setting where patients could refuse hospital treatment so extrapolation of this information is of limited relevance. The high number of SPs with schizophrenia still admitted may be because of the chronicity of the illness and associated severe functional impairment. Patients with schizophrenia are less likely to have insight into their illness or their offences. Schizophrenia has been associated with long-term psychiatric care.^15^ SPs at Sterkfontein Hospital presented with a distribution of psychiatric illnesses similar to this study, although almost 70% of offenders were back in the community after 3 years.^9^

At Weskoppies Hospital, SPs who had been admitted for 36 months (3 years) or less were more common in the AG, while patients who had been admitted for 109–144 months (9–12 years) were more common in the DG. SPs in the first 3 years of their admission may not yet have responded to treatment or achieved adequate control of the symptoms of their illnesses. SPs who were admitted for longer possibly had more exposure to therapeutic interventions, increasing the likelihood of their illness being stabilised. SPs admitted for a longer period are also monitored for longer periods. High-risk behaviours may decrease or disappear over time, increasing the likelihood of discharge. Stay in semi-open wards between 3 and 6 years was associated with continued admission, while prolonged stay in open wards (6–9 years) was associated with discharge. Patients in access-controlled wards are more likely to exhibit high-risk behaviours resulting in continued hospitalisation. Patients in open wards have more opportunities for integration into the community through LOAs from the hospital. LOAs are granted if high-risk behaviour is absent or has decreased. The number of successful LOAs was significantly associated with discharge. Patients who are granted LOAs are assessed as displaying reduced risk behaviour related to possible future unlawful acts.

The absence of deviant behaviours (sexually inappropriate behaviour, physical or verbal aggression and continued substance use) during admission was associated with discharge. In contrast, recent aggression (verbal or physical) and recent substance abuse were associated with continued admission. These are high-risk behaviours which, when present, may lead to prolonged admission. Most studies focus on identifying factors that may lead to aggressive behaviour,^16^ rather than determining if these factors are associated with prolonged admission. We propose using aggressive behaviour as a predictive factor for continued admission.

Our study has several limitations. Firstly, because of extended periods of admission and the hospital being an academic institution, patient notes were often recorded by different health-care practitioners rotating through the forensic unit. This could have led to inconsistent record-keeping. Secondly, patients often had several hand-written files that increased the possibility that not all notes were available for review. We excluded patient records that were grossly incomplete, but other data gaps may have been present. We did not quantify the relationship between various factors. Relationships between behavioural factors, psychiatric factors and criminal factors were not explored. Although the profiles of the patients are consistent with those of other local studies,^9,10^ they may not be generalisable or applicable to different hospital settings.

## Conclusion

The number of patients entering the forensic system has increased steadily both locally and internationally. This places strain on the resources of an already overburdened system. There is a need for shortened inpatient length of stay without compromising the safety of the community or the patients.

Our study identified factors associated both with discharge and continued admission. SPs who are primary care givers, are not diagnosed with a substance use disorder, have good insight into their illness and offence, and have no previous offences were more frequently discharged. At Weskoppies Hospital, SPs who were admitted for between 9 and 12 years and granted multiple successful LOAs were also more frequently discharged. Absence of high-risk behaviour such as substance abuse, and verbal and physical aggression were also associated with being discharged.

In contrast, short length of total stay (< 3 years), prolonged stay in semi-open wards and short length of stay in open wards were associated with continued admission. Absence of successful LOAs, history of previous offences, lack of insight into illness and offence were also associated with continued admission. Furthermore, the presence of high-risk behaviours (as noted in the previous paragraph) was associated with continued admission. This study highlights specific factors that should be considered during risk assessments of SPs who are being considered for possible discharge.
