# Erratum: Identifying factors associated with the discharge of male State patients from Weskoppies Hospital

**DOI:** 10.4102/sajpsychiatry.v25i0.1353

**Published:** 2019-10-10

**Authors:** Riaan G. Prinsloo, Andre Swanepoel, Gian Lippi

**Affiliations:** 1 Department of Psychiatry, School of Medicine, Faculty of Health Sciences, University of Pretoria, South Africa; 2 Department of Statistics, Faculty of Natural and Agricultural Science, University of Pretoria, South Africa

In the author list of this article published earlier, Gian Lippi’s ORCID was unintentionally misprinted as http://orcid.org/0000-0002-9545-240X.

The correct ORCID for Gian Lippi is http://orcid.org/0000-0002-1214-3573. The publisher sincerely regrets this error and apologises for any inconvenience caused.

